# Neoadjuvant Therapy in Lung Cancer: What Is Most Important: Objective Response Rate or Major Pathological Response?

**DOI:** 10.3390/curroncol28050350

**Published:** 2021-10-13

**Authors:** Xi Chen, Kewei Ma

**Affiliations:** Cancer Center, The First Hospital of Jilin University, 71 XinMin Street, Changchun 130021, China; chenxi7017@mails.jlu.edu.cn

**Keywords:** non-small cell lung cancer, neoadjuvant therapy, objective response rate, major pathological response

## Abstract

Lung cancer is the most fatal and frequently diagnosed malignant tumor. Neoadjuvant therapy is a promising approach for prolonging survival and increasing the chance of cure rates for patients with potentially resectable disease. Currently, many therapeutic alternatives, including chemotherapy, targeted therapy, and immunotherapy, are continually being explored to enrich the content of neoadjuvant therapy. However, neoadjuvant therapy remains to have no unified evaluation standards. Overall survival (OS) is the “gold standard” for evaluating the clinical benefit of cancer treatment, but it needs years for a reliable evaluation. Hence, researchers need to identify surrogate endpoints that can predict OS accurately and reliably without long follow-up periods. In this review, we describe the research progress of different neoadjuvant therapies and explore their response evaluation, aiming to identify stronger predictors of OS.

## 1. Introduction

Lung cancer, especially non-small-cell lung cancer (NSCLC), which represents 80–85% of all cases [1], remains to be a lethal cancer type worldwide despite tremendous advances in treatment strategies [2]. In patients with resectable NSCLC, surgical resection combined with systemic adjuvant therapy leads to poor overall survival (OS) because microscopic distant metastases generally occur in these patients. The 5-year survival rate is approximately 77–92% at stage I and then decreased to 53–60% and only 36% at stages II and IIIA, respectively [3].

For patients with early-stage disease, complete surgical resection is still the most effective initial therapy [4]. Neoadjuvant therapy is an accepted practice in patients with operable and locally advanced lung cancer. For patients with NSCLC at stage I to II, neoadjuvant therapy could eliminate all micrometastasis to the greatest extent, thereby increasing the chance of survival. For patients with locally advanced NSCLC (stage IIIA), it might downstage the tumors and make tumors more operable, potentially increasing the chance of performing complete resection. Presently, various neoadjuvant therapy options, such as chemotherapy [5], chemoradiotherapy [6], targeted therapy [7], and immunotherapy [8], are available. However, neoadjuvant therapy remains to have no unified evaluation standards, and the most important factor affecting long-term survival after treatment remains debatable.

From a large number of clinical neoadjuvant chemotherapy and targeted therapy, no significant improvement of OS was found. These unsatisfactory results caused our attention and we began to think about how to effectively evaluate the impact of neoadjuvant therapy on survival. Impressed by the great improvement of major pathological response (MPR) rates in neoadjuvant immunotherapy, we reviewed the MPR results from previous neoadjuvant chemotherapy and targeted therapy, and concluded that low MPR may be the reason for unsatisfactory OS. Thus, we compared the radiographic response and pathological response in different neoadjuvant therapy. In this review, we aimed to discuss which indicator has a more predictive value for OS in different neoadjuvant therapies and to explore the most promising treatment for potentially resectable NSCLC.

## 2. Methods

We performed a literature search focusing on outcomes of neoadjuvant therapy in patients with resectable NSCLC. We explored PubMed and Google Scholar with the following search terms: (1) non-small cell lung cancer (NSCLC); (2) neoadjuvant; (3) chemotherapy; (4) targeted therapy; (5) immunotherapy; (6) objective response rate (ORR); and (7) major pathological response (MPR). There was no restriction in article type. We reviewed relevant articles independently and summarized the results of various studies. In this review, we have included the most up-to-date information of neoadjuvant therapy and predictor of OS in resectable NSCLC.

## 3. Correlation between Complete Resection and OS

Complete resection is an important predictor of OS, but is affected by objective response rate (ORR) and major pathological response (MPR). Preoperative therapy is important because it might downstage the malignancy and make tumors more operable, thereby potentially increasing the chance of performing complete resection. Complete resection is an important factor for predicting OS, which is a widely recognized endpoint and a “gold standard” in determining clinical benefits from any oncology trials. Patients undergoing complete resection can obtain a median OS of 80.1 months and a 5-year survival rate of 58.8% [9]. After neoadjuvant therapy, the rate of complete resection reportedly highly improves. However, a question was raised as to whether or not performing complete resection could actually leave no residual tumor and bring long-term survival.

Table 1 summarizes the results of phase III clinical trials evaluating neoadjuvant chemotherapy. Various trials showed that the increase of complete resection rate does not necessarily cause a high OS to the same degree. This finding can also mean that complete resection may be closely associated with a better prognosis, but for patients with locally advanced NSCLC, the prognosis depends more on the presence of residual micrometastases. Although complete resection may be successfully done through preoperative downstaging, the presence of undetected micrometastases could lead to a great risk for recurrence and poor survival. Therefore, using the rate of complete resection as an index is inappropriate to assess the efficacy of neoadjuvant therapy. Exploring the most important predictor for postoperative survival is necessary to assess efficiency after treatment. This factor might be the ORR, MPR, or pathological complete response (pCR).

## 4. ORR, Related with OS but Not Tightly

The tumor ORR is the proportion of patients achieving a complete response (CR) or a partial response (PR) evaluated by Response Evaluation Criteria in Solid Tumors (RECIST) criteria version 1.1. This rate is used for assessing tumor burden after providing chemotherapy treatment to patients with solid tumors. However, with the emergence of immunotherapy, researchers question whether RECIST criteria (the anatomic response criteria reflecting tumor shrinkage mainly for cytotoxic chemotherapy) applies to immunotherapy as well.

The correlation between ORR and OS. Several studies have already investigated whether changes in tumor volume determined from radiological evaluations are closely associated with survival in patients with NSCLC undergoing neoadjuvant chemotherapy. Birchard et al. reviewed 99 patients and concluded that radiological response had no correlation with survival (*p* = 0.754) and that survival was not significantly different between patients with initial disease progression and those with an initial reduction in tumor size (*p* = 0.580) [21].

Additionally, a phase III trial by Gilligan et al. explored survival outcomes of 519 patients received surgery alone or platinum-based chemotherapy preoperatively. The neoadjuvant chemotherapy group showed a positive ORR (49%) [15]. However, the 5-year OS did not improve (44% in the neoadjuvant chemotherapy group vs. 45% in the surgery alone). Likewise, another trial conducted by Depierre demonstrated no statistical significance in 4-year survival rates between patients received preoperative chemotherapy and surgery alone. Hence, neoadjuvant chemotherapy could lead to great remission on imaging, but relevant survival outcomes were poor.

Better ORR can decrease tumor burden, but not closely associated with OS. Table 2 lists the results of phase II clinical trials of neoadjuvant-targeted therapy. The neoadjuvant-targeted treatment tended to have a higher ORR than neoadjuvant chemotherapy. In a systematic review of five clinical trials involving 124 patients receiving the neoadjuvant erlotinib or gefitinib, the pooled ORRs of all patients in stage I–IIIA and those in stage IIIA subgroup were 58.5% and 51.4%, respectively; both results were numerically superior to those in previous neoadjuvant chemotherapy trials. The pooled median progress-free survival (PFS) was 13.2 months. The surgical resection and R0 rates in stage IIIA-N2 subgroup were 79.7% and 56.8%, but the downstaging and pCR rates were merely 14.0% and 0%, respectively, which were numerically lower than those in previous studies of neoadjuvant chemotherapy [22]. In the CTONG 1103 trial, the benefits of erlotinib were compared with those of gemcitabine plus cisplatin as neoadjuvant therapy. The erlotinib group achieved a higher ORR (54.1% vs. 34.3%), but both groups had unimpressive MPR rates (9.7% vs. 0%) and the OS was not significantly different between the two groups (45.8 months vs. 39.2 months) [7].

Therefore, neoadjuvant-targeted therapy could significantly shrink tumor volume and improve radiological responses, as well as increasing the curative resection rate. However, this impressive tumor shrinkage effect has not been translated into changes in disease stage or pCR rate. Additionally, the insight into whether neoadjuvant-targeted therapy could improve OS compared with neoadjuvant chemotherapy remains unclear. 

The advent of neoadjuvant immunotherapy has revolutionized the treatment landscape of metastatic and stage III NSCLC. In a phase II pilot single-arm study by Forde, after two preoperative doses of nivolumab, the ORR was only 10%, but with an unexpectedly higher MPR rate of 45% (9 of 20). The rate of recurrence-free survival (RFS) at 18 months was 73%. Considering the relatively short follow-up time, the median OS duration was not reached [8]. Nevertheless, the current prognosis was impressive, worth looking forward to long-term effectiveness. Additional investigation is required to determine whether a high MPR rate after neoadjuvant immunotherapy could further contribute to longer PFS or even OS.

As observed in the abovementioned trials, neoadjuvant chemotherapy and neoadjuvant-targeted therapy could sharply increase ORR but could not show significant improvement in OS. In contrast, despite low ORR, the survival outcomes after neoadjuvant immunotherapy were extremely promising. Hence, as the duration of treatment and treatment-related interventions increases, the ORR is not always reliable in predicting corresponding effects in OS, and the ORR assessed by RECIST1.1 is better used only for cytotoxic chemotherapy.

## 5. Is MPR Better Than ORR?

MPR is tightly correlated with OS. MPR refers to the presence of no >10% viable tumor cells in the resection specimen. The potential of MPR as a surrogate for OS had gained research interest. Junker et al. retrospectively explored the correlation between histopathological regression and survival outcomes through a thorough pathological analysis of 40 patients who underwent neoadjuvant bimodality treatment. The median survival time was statistically significantly longer in the cohort that achieved MPR (36 months) than in all other cohorts with >10% residual tumor (14 months) (*p* = 0.02) [28], indicating that people achieving MPR may have a better survival prognosis.

Moreover, Pataer assessed the capacity of histopathological response to predict a long-term outcome in patients treated with or without neoadjuvant chemotherapy. Results revealed that the proportion of residual viable tumor cells strongly correlated with OS and disease-free survival (DFS) in patients with neoadjuvant chemotherapy regardless of being controlled for pathologic stage. Patients who had <10% viable tumor had significantly longer OS and DFS than those with >10% viable tumor cells (5-year OS, 85% vs. 40%; 5-year DFS, 78% vs. 35%) [29]. Hence, a large number of clinical research data have demonstrated that MPR has a strong correlation with OS.

Discrepancy between radiographic response and pathological response. Presently, between MPR and ORR, the insight into which is better to appraise the efficacy of neoadjuvant therapy remains controversial. The trend of MPR and ORR was inconsistent among different trails as shown in Table 2 and Table 3. Taking the CheckMate-159 trial for example, despite low ORR, the MPR rate (45%) was unexpectedly higher [8]. As shown in an extended follow-up report, the RFS at 24 months was 69%, and 75% of patients remained alive at 30 months [30]. Though this study was limited by a short follow-up period without abundant survival data, the intermediate-term prognosis is favorable. These results suggested that when ORR and MPR are quite different in number, MPR may have a more important effect on OS.

A previous study investigated whether or not tumor response based on CT scan findings using the RECIST criteria could predict OS and histopathological response in patients treated with neoadjuvant chemotherapy. The discordance rate was 41% between histopathological response and radiographic response. The histopathological response was proven to be a stronger predictor of OS than radiographic response (*p* = 0.002) than CT-measured tumor response (*p* = 0.03). In patients who had completed neoadjuvant chemotherapy with >10% remaining viable tumor, the survival outcomes of patients with PR or CR were not significantly different from patients with progressive disease (PD) or stable disease (SD) [31]. Thus, the radiographic response may not be reliably predictive in all patients because it cannot identify patients who have pathological remission. Conversely, the pathological response may be the most vital predictor of OS.

Among various measurements of pathological response, pCR has been preferred for decades. Although pCR is associated with longer survival, it is restricted by its infrequency; the occurrence of pCR in neoadjuvant chemotherapy is generally <10%. The different responses of the primary tumor and mediastinal lymph node metastasis to systemic chemotherapy cause an out-of-sync pathological remission. The nodal response is also associated with long-term survival, but it relies on the accuracy of nodal assessment and is only applicable to patients with lymph node disease confirmed by pathology during diagnosis [32]. Conversely, MPR, which is applicable to disease at all stages, and is independent of preoperative staging accuracy, is ideally suited as an alternative indicator of survival in patients with NSCLC who underwent surgery after neoadjuvant therapy. 

**Table 3:** Phase II clinical trials of neoadjuvant immunotherapy.

Trial	Stage	Size	Intervention Used	ORR	MPR	pCR	Survival
CheckMate-159 (NCT02259621) [8]	IB–IIIA	22	Nivolumab	10%	45%	10%	RFS at 18 months: 73%
LCMC3 (NCT02927301) [33]	IB–IIIB	181	Atezolizumab	7%	20.4%	6.8%	OS at 12 mo: 92% (stage II) 95% (stage III)
NEOSTAR (NCT03158129) [34]	I–IIIA	37	Nivolumab + Ipilimumab vs. Nivolumab	NI: 19%, N: 19%	NI: 50%, N: 24%	NI: 38%, N: 10%	NR
ChiCTR-OIC-17013726 [35]	IA–IIIB	40	Sintilimab	NR	40.5%	16.2%	NR

ORR, objective response rate; MPR, major pathological response; pCR, pathological complete response; OS, overall survival.

## 6. Reasons Accounting for the Difference between ORR and MPR

The numerical differences across trials likely reflect differences between histopathological response and radiographic response assessed by RECIST criteria. Knowing the reason for these discrepancies could further help identify significant predictors of OS. The major factor driving this inability of tumor size changes assessed by conventional imaging to predict pathological remission is the composition of NSCLC tumors, which contain a pathologically heterogeneous mixture of cancer cells, stromal tissue, and associated inflammatory cells. Despite that gross necrosis can be identified by CT, its features of stromal, inflammatory, or fibrotic alterations resemble those of viable tumor cells. Consequently, RECIST-assessed radiographic response may only evaluate primary tumor macroscopically because of a high likelihood that the tumor size changes on imaging can be easily confounded by inflammatory or fibrotic changes and difficult to reflect the real remission.

This confusion is magnified by a special immune killing process after neoadjuvant immunotherapy. By interrupting the programmed death protein 1 (PD-1)/programmed cell death 1–ligand 1 (PD-L1) regulatory axis, cytotoxic T lymphocyte antigen-4 (CTLA-4), or others, immune checkpoint inhibitors (ICIs) can initiate T cell activation and expansion, increasing the quantity of activated tumor-specific CD8+ T cells. CD8+ T cells could not only kill tumor cells directly but also release more new tumor antigens; subsequently, new antigens released from the collapsed tumor cells are delivered to specific effector T cells at different sites, further amplifying the immune response. Thus, compared with chemotherapy and molecular-targeted therapy, neoadjuvant immunotherapy results in the infiltration of considerably numerous T cells in tumor specimens; hence, the inflammatory reaction is prolonged. The necrotic tumor tissue, which is only surrounded by new connective tissues, is difficult to dissolve and absorb. Progression identified on imaging may be actually a downsized tumor with appropriate infiltration of immune cells, referring to “pseudoprogression” [36]. Taken together, different treatment methods, such as anti-PD-L1 therapy and chemotherapy, may lead to differences in the pathological changes within the tumor, making the evaluation of the efficacy only by changes on imaging in clinical practice more difficult and causing the different changes between ORR and MPR.

## 7. Clinical Value of MPR in Neoadjuvant Immunotherapy

MPR can help catch the best timing of surgery. The aforementioned difference between ORR and MPR is crucial in clinical practice. Using only ORR to evaluate tumor shrinkage and antitumor activity may significantly underestimate the benefits derived from neoadjuvant therapy especially immunotherapy, and it may even miss the best timing of surgery. In the NADIM trial, 3 (33%) of 9 patients with SD and 22 (73%) of 30 patients with a PR demonstrated a pCR instead [37]. After only 2–4 cycles of preoperative immunotherapy, being assessed as SD may greatly underestimate the true efficacy because of the worsening infiltration of lymphocytes, and while making the SD assessment, patients may have already achieved MPR or even pCR. A previous study revealed that in 28 patients suffering from glioblastoma multiforme with pseudoprogression confirmed by salvage pathologies, 12 (42.8%) experienced unnecessary surgery risk because their tumors were misclassified as true tumor progression by magnetic resonance imaging (MRI) [38]. 

Despite various methods of assessing MPR being used, they have not been defined in detail. In 2020, the International Association for the Study of Lung Cancer (IASLC) has made detailed recommendations on how to assess pathological response of lung cancer resection specimens after neoadjuvant therapy. A standardized approach is recommended to assess the percentages of (1) viable tumor, (2) necrosis, and (3) stroma (including inflammation and fibrosis) with a total adding up to 100%, which can be used for all systemic therapies [39]. 

Before surgery and consideration of neoadjuvant therapy, staging with 18F-fluorodeoxyglucose positron-emission tomography (PET) scan is recommended. Different from traditional radiologic imaging technologies, which are based on gross anatomical changes to provide structural information and define disease states, PET imaging provides information on the biochemical processes that may precede gross anatomic change [40]. In the ChiCTR-OIC-17013726 trial, they introduced baseline and preoperation PET–CT to evaluate tumor response and recorded maximum standardized uptake value (SUVmax) to estimate the change of tumor activity in neoadjuvant immunotherapy. A significant correlation between SUVmax reduction and pathologic response was found, indicating that SUVmax might help to assess tumor pathologic response before operation and catch the best timing of surgery [35].

MPR rate was sharply increased in neoadjuvant immunotherapy. As mentioned above, MPR after neoadjuvant therapy could predict the OS of patients with NSCLC. By comparing the rate of MPR among different neoadjuvant approaches, we may determine the most efficient treatment region. Many previous studies used MPR as the primary or secondary endpoint. Results showed that after neoadjuvant chemotherapy, the MPR rates were generally between 7% and 27% [29,41,42,43,44], and adding radiotherapy was not beneficial [6]. Molecular-targeted therapy could not considerably improve the MPR rate as well. 

To our satisfaction, since the advent of immunotherapy, particularly ICIs, numerous breakthroughs have been achieved, with new results emerging exponentially. Although vital clinical trials are still ongoing and more data are needed, the completed trials have reported stimulating results. As shown in Table 3 and Table 4, the MPR rate of immune monotherapy reached 22–45%, whereas that of immunochemotherapy could reach as high as 36.9–83% with encouraging survival outcomes. These results demonstrate convincingly that neoadjuvant immunochemotherapy is the most established and clinically promising therapy for patients with potentially resectable NSCLC.

Overall, MPR plays a more important role in long-term survival, and it could predict OS, serving as a surrogate endpoint. In neoadjuvant immunotherapy, the MPR rates were sharply increased. Although vital clinical trials are still in progress, results obtained from the completed trials were highly encouraging. Evaluating MPR after neoadjuvant immunotherapy with the help of PET is necessary to grasp the best timing of surgery.

## 8. Conclusions

MPR may be a more decisive factor of long-term OS in patients with NSCLC than ORR. It could act as an effective surrogate endpoint to characterize the activity of neoadjuvant chemotherapy, targeted therapy, and immunotherapy. As discussed above, previous neoadjuvant chemotherapy has dramatically increased ORR, but PFS and OS were not significantly extended; neoadjuvant targeted therapy produced better ORR but poor MPR and survival; neoadjuvant immunotherapy has sharply increased MPR with encouraging interim survival results, and adding chemotherapy would improve ORR as well. Therefore, neoadjuvant immunotherapy is an optimal treatment strategy for potentially resectable NSCLC. The paradigm of NSCLC treatment is expected to be drastically changed by neoadjuvant immunotherapy alone or in combination in the not-too-distant future. Considering that the traditional RECIST criteria cannot appraise the efficacy of immune-related therapy more accurately, accurate evaluation of MPR after neoadjuvant immunotherapy is crucial and staging with PET is recommended. Despite the potential interest of MPR as a surrogate of OS, the specific value has not been established yet, and further studies are needed. A new pathological evaluation standard should be developed, which is applicable to all current treatment methods. 

## Figures and Tables

**Table 1:** Phase III clinical trials of neoadjuvant chemotherapy.

Trial	Size	Stage	Histology No. (%)	Regimen	ORR	pCR	Complete Resection Induction Chemo vs. Surgery Alone	Median OS Induction Chemo vs. Surgery Alone	Survival Induction Chemo vs. Surgery Alone
Roth [10]	60	IIIA	AD: 30(50) SCC: 22(37) LCC: 6(10)	Cyclophosphamide Etoposide Cisplatin	35%	NR	39% vs. 31%	64 months vs. 11 months *	OS at 36 months 56% vs. 15%
Rosell [11,12]	60	IIIA	AD: 14(23) SCC: 42(70) LCC: 4(7)	Mitomycin Ifosfamide Cisplatin	60%	4%	85%	22 months vs. 10 months ^†^	OS at 60 months 17% vs. 0%
Depierre [13]	355	IB–IIIA	AD SCC	Mitomycin Ifosfamide Cisplatin	64%	11%	92% vs. 86%	37 months vs. 26 months ^‡^	OS at 48 months 43.9% vs. 35.3%
Nagai [14]	62	IIIA	AD: 41(66) SCC: 15(24) Others: 6(10)	Cisplatin Vindesine	28%	0%	65% vs. 77%	17 months vs. 16 months ^§^	OS at 60 months 10% vs. 22%
Gilligan [15]	519	IB–IIIA	AD: 138(27) SCC: 256(49) Others: 125(24)	Platinum-based	49%	4%	82% vs. 80%	54 months vs. 55 months **	OS at 36 months 44% vs. 45%
Pisters [16,17]	354	IB–IIIA	AD: 107 SCC: 129 Others: 101	Paclitaxel Carboplatin	41%	NR	93% vs. 88%	62 months vs. 41 months ^††^	OS at 60 months 50% vs. 41%
Felip [18]	413	IB–IIIA	AD: 128(31) SCC: 212(52) LCC: 42(10) Others: 27(7)	Paclitaxel Carboplatin	53.3%	10.5%	NR	NR	OS at 60 months46.6% vs. 44% II-T3N1: 41.3% vs. 34.5%
Scagliotti [19]	270	IB–IIIA	AD: 85(31) SCC: 111(31) LCC: 13(1) Others:59(22)	Gemcitabine Cisplatin	35.4%	NR	88% vs. 84%	93 months vs. 57 months ^‡‡^	OS at 36 months67.6% vs. 59.8% SCC: 66.5% vs. 65.6%
Mattson [20]	274	IIIA- IIIB	AD: 54(20) SCC: 170(62) LCC: 20(7) Others:30(11)	Docetaxel	28%	NR	77% vs. 76%	14.8 months vs. 12.6 months ^§§^	OS at 12 months 59.1% vs. 50.5%

* *p* = 0.008 by logrank test and *p* = 0.018 by Wilcoxon test; ^†^ 22 months (95% CI, 13.4–30.6) vs. 10 months (95% CI, 7.4–12.6; *p* = 0.005); ^‡^ 37 months (95% CI, 26.7–48.3) vs. 26 months (95% CI, 19.8–33.6; *p* =0.15); ^§^ *p* = 0.5274, not significant; ** HR 2.01, 95% CI 0.80–1.31, *p* = 0.86, not significant; ^††^ HR 0.79, 95% CI 0.60–1.06, *p* = 0.11; ^‡‡^ HR 0.63, 95% CI, 0.43–0.92, *p* = 0.02; ^§§^ not significant; ORR, objective response rate; OS, overall survival; NR, not reported; AD, adenocarcinoma; SCC, squamous cell carcinoma; LCC, large cell carcinoma.

**Table 2:** Phase II clinical trials of neoadjuvant-targeted therapy.

Trial	Stage	Size	Intervention Used	ORR	Complete Resection	MPR	pCR	Survival
CTONG1103 [7]	IIIA, N2	72	Erlotinib vs. Gemcitabine + Cisplatin	54.1% vs. 34.3%	73% vs. 62.9%	9.7% vs. 0%	0% vs. 0%	mPFS: 21.5 months vs. 11.4 months mOS: 45.8 months vs. 39.2 months *
Zhang, Y. [23]	II- IIIA	33	Gefitinib	54.5%	NR	24.2%	NR	mDFS: 33.5 months OS at 48 months: 54.5%
Xiong, L. [24]	IIIA	19	Erlotinib	42.1%	68.4%	NR	NR	mOS: 51.6 months
Lv, C. [25]	I–IIIA	134	EGFR-TKI vs. Pemetrexed + Cisplatin	55.8% vs. 38.5%	95.3% vs. 95.6%	NR	0% vs. 2.2%	mDFS: 15.0 months vs. 14.1 months ^†^ OS at 36 months: 76.6% vs. 66.8%
ASCENT [26]		19	Afatinib + CRT	69%	NR	57.1%	14.3%	OS at 24 months: 85% mPFS: 34.6 months
Bao, Y. [27]	IB-IIIC	42	EGFR-TKIs	47.6%	NR	23.8%	NR	mRFS: 19.8 months

* HR, 0.77; 95% CI, 0.41–1.45; *p* = 0.417, not significant; ^†^ 0.895 95% CI, 0.402–1.993; *p* = 0.871 not significant; EGFR-TKI, epidermal growth factor receptor–tyrosine kinase inhibitor; ORR, objective response rate; MPR, major pathological response; mPFS, median progress-free survival; mRFS, median recurrence-free survival; mDFS, median disease-free survival; OS, overall survival; CRT, chemoradiotherapy; NR, not reported.

**Table 4:** Phase II clinical trials of neoadjuvant immunochemotherapy.

Trial	Stage	Size	Intervention Used	ORR	MPR	pCR	Survival
NADIM (NCT03081689) [37]	IIIA, N2	46	Nivolumab + Paclitaxel, carboplatin	78%	83%	71%	OS at 24 months: 89.9%
TOP1201 (NCT01820754) [45]	IB–IIIA	24	Ipilimumab (cycles 2–3 only) Paclitaxel Cisplatin (or carboplatin)	58%	NR	15%	OS at 24 months: 73.0%
MAC (NCT02716038) [46]	IB–IIIA	30	Atezolizumab + Nab-paclitaxel, carboplatin	63%	57%	33%	mDFS: 17.9 months
CheckMate816 (NCT02998528) [47]	IB–IIIA	350	Chemotherapy + nivolumab vs. chemotherapy	NR	36.9% vs. 8.9%	24% vs. 2.2%	NR
Duan, H. [48]	IIA–IIIB	23	Chemotherapy + PD-1 inhibitor	73.9%	50%	30%	mPFS: 11.3%
Shen, D. [49]	IIB–IIIB	37	Chemotherapy + pembrolizumab	86.5%	64.9%	45.9%	NR

ORR, objective response rate; MPR, major pathological response; pCR, pathological complete response; mDFS, median disease-free survival; OS, overall survival; PD-1, programmed cell death protein 1.
